# Understanding Global Cancer Disparities: The Role of Social Determinants from System Dynamics Perspective

**DOI:** 10.22545/2016/00072

**Published:** 2016-04-10

**Authors:** Faustine Williams, Nancy Zoellner, Peter S. Hovmand

**Affiliations:** 1 Division of Public Health Sciences, Department of Surgery, Washington University in St. Louis School of Medicine, St. Louis, USA, WilliamsF@wudosis.wustl.edu; 2 Social System Design Lab, George Warren Brown School of Social Work, Washington University in St. Louis, St. Louis, MO USA.

**Keywords:** Global health, cancer disparities, social determinants, system dynamics, systems science, global development, economic development

## Abstract

**Background:**

In 2012, almost 57% of all cancer cases and 65% of cancer deaths occurred in low-and middle-income countries. If the current trend continues, the burden of cancer will increase to 22 million new cases annually by 2030, with 81% of new cases and almost 88% of mortality occurring in less developed countries.

**Methods:**

A qualitative review of the literature was conducted. This included a systematic search of eight electronic databases namely, PubMed, Academic Search Premier, CINAHL, Applied Social Sciences Index, EMBASE, SCOPUS, Cochrane and PsycINFO. The reference list of articles retrieved were also thoroughly searched. Inclusion criteria were studies that addressed global health, cancer disparities and global or economic development.

**Results:**

Thirty-one articles were identified that met the eligibility criteria. Results were synthesized in the form of a system dynamics causal loop diagram or map which led to identification of eight major stocks or system variables. These included, children and adult population, overall population health, pollution, quality of healthcare delivery, quality of neighborhood and built environment, social and community cohesiveness, healthy and social norms and attitudes, and literacy level. Based on this, a dynamic hypothesis of global health cancer disparities was developed. The causal loop diagram showed the role of multiple interacting feedback mechanisms as explanations for trends in global health cancer disparities and the underlying consequences.

**Conclusions:**

Addressing these determinants of health requires an effective dynamic approach to improving global cancer health. Application of a systems thinking methodological approach has the potential to provide new understanding to how global development trends in combination with global health efforts to improve population health could shift cancer disparities and burden associated with the disease.

## 1 Introduction

**Figure 1:**
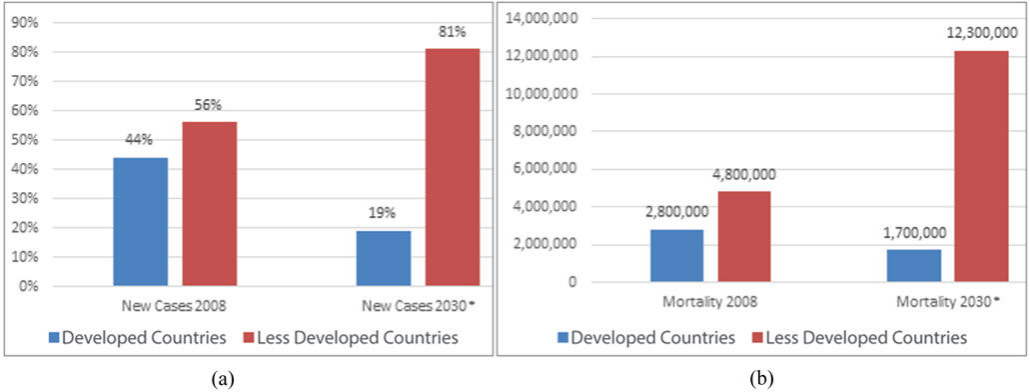
(a) Estimated new cancer cases for 2008 and 2030, (b) Estimated Cancer Deaths for 2008 and 2030

Cancer is the leading cause of death globally, costing the world economy almost one trillion dollars per year [1]. The total economic impact of premature death and disability from cancer worldwide was $895 billion in 2008, 20% higher than heart disease [1]. According to the International Agency for Research on Cancer, 8.2 million cancer related deaths occurred globally in 2012, compared with 7.6 million in 2008 [2]. Of these, almost 57% of all cancer cases and 65% of cancer deaths occurred in low-and middle-income countries (LMCs). If the current trend continues, the burden of cancer will increase to 22 million new cases annually by 2030, an increase of almost 70% from 2008 [2]. Worldwide cancer deaths are also projected to continue to rise to approximately 13.2 million in 2030 due to population growth and aging [3-6]. The diagrams show the global distribution of new cancer diagnoses and projected deaths for 2008 and 2030 (see Figure 1 a & b).

Cancer mortality in most developed countries has been decreasing since the mid-1990 due primarily to advances in biomedical technology leading to an increase in early diagnosis and treatment [7,8]. However, the impact on individuals, communities and populations constitutes a major threat to advancement in many less developed countries, due to lack of access to healthcare services, poverty, and education, all of which increases morbidity and mortality from the disease [1,2,9,10].

Given the complex set of interactions of social determinants underlying global health, especially cancer disparities, social and economic development, as well as population health, new methods are needed to better identify and understand the potential feedback mechanisms driving long-term trends in development, social determinants, and cancer deaths. One such method, system dynamics, studies complex, nonlinear feedback systems and their dynamics [11]. Prior work exists on specific diseases and healthcare delivery, but there has been little application of system dynamics to global cancer disparities.

### 1.1 Aim

The objective of this work was to develop a conceptual framework in the form of a system dynamics causal map based on the extant literature of the systems underlying trends in global cancer disparities.

## 2 Approach and Methods

### 2.1 Systematic Review Process

A qualitative review of the literature was conducted. This included a systematic search of PubMed (1951-2013), Academic Search Premier (1984-2013), CINAHL (1937-2013), Applied Social Sciences Index and Abstracts (1987-2013), EMBASE (1947-2013), SCOPUS (1982-2013), Cochrane (1993-2013) and PsycINFO (1987-2013) databases using the keywords: “global health”, OR “economic development” OR “human development” AND “cancer “disparities” OR “inequalities”, AND “social determinants” OR “social determinants of health” AND “system dynamics” “systems thinking” OR “system science”. In addition, secondary references were retrieved within the reference lists of publications that were included for review. There was no limitation of publication date in the search; however, the earliest eligible article was published in 1993. Inclusion criteria were studies that addressed global health, cancer disparities and global or economic development and published in the English language. Studies not meeting these criteria were excluded.

## 3 Results

### 3.1 Global Cancer Disparities Concept Model Scope and Subsystems

**Figure 2:**
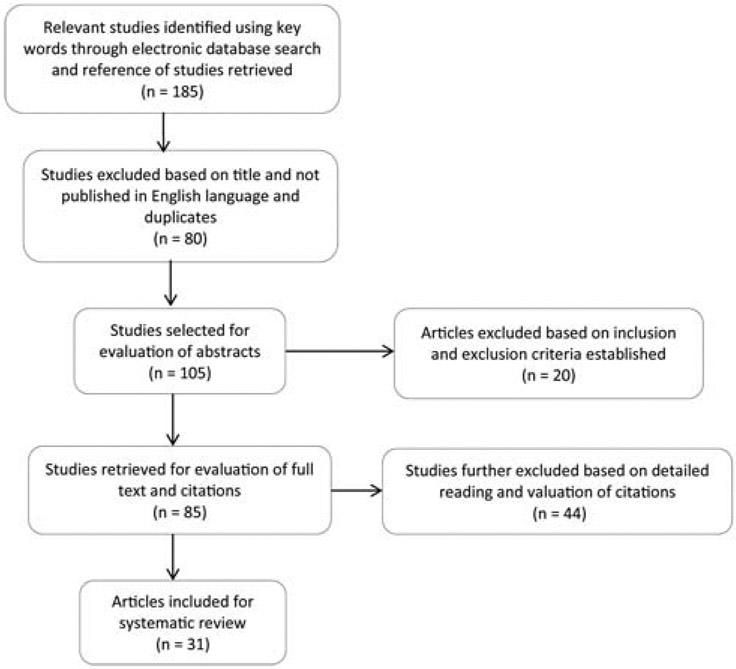
Flow Diagram for literature search results and application of eligibility criteria

A total of 183 original full-text articles were found across the electronic databases mentioned earlier, and 2 additional studies were identified through references of articles retrieved. Eighty studies that were either duplicates, and not published in the English language were further eliminated. Based on the inclusion and exclusion criteria established 20 studies were removed. Full screening was performed on the remaining 85 studies. After thorough review 44 studies were further excluded. A total of 31 [4-6,8,10,12-37] studies were considered eligible and used in the development of model scope and causal loop diagram or map. The flow chart, shows the summary of criteria used for inclusion of eligible studies (see Figure 2).

**Figure 3:**
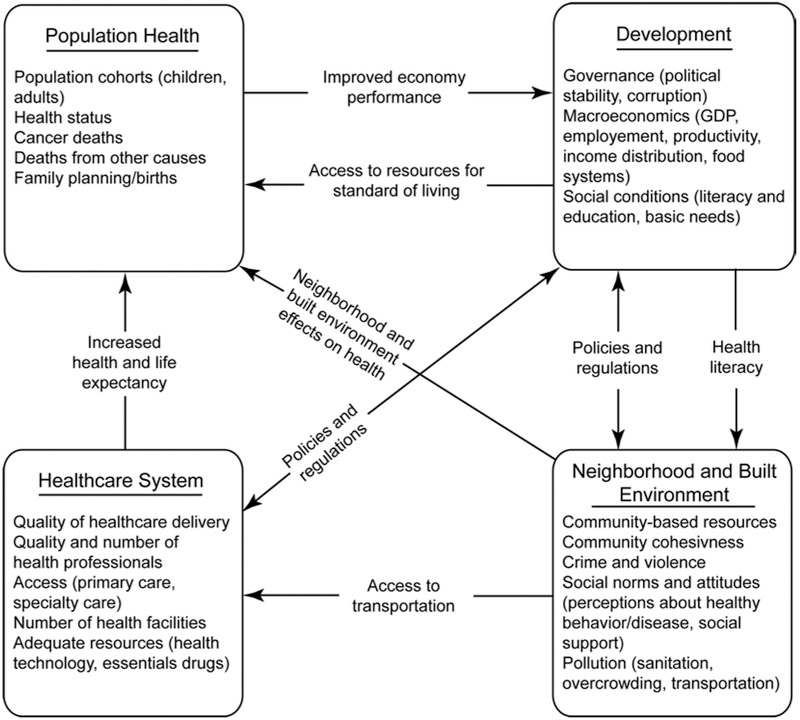
Model scope and subsystem diagram

The resulting model scope consists of four main sectors or subsystems: population health, development, healthcare system and neighborhood and built environment (see subsystem diagram in Figure 3). The model scope and subsystem diagram (Figure 3) illustrates how healthcare, environment, governmental policies and overall level of development collectively shape and influence population growth, inequalities in healthcare and health outcomes.

Countries in Sub-Saharan Africa are already struggling to control the widespread of many communicable/tropical diseases facing them. The emergence of non-communicable diseases such as cancer is likely to exacerbate the public health problems. The population health subsystem (Figure 3: top left) captures the number of adults, children, new births as well as mortality and morbidity from cancer and other diseases. The level of population health is influenced by the country’s economic development and resources available to promote better quality of living. For instance, lower mortality from diseases will lead to significant increase in workforce of a country thereby improving economic performance. Additionally, healthy workforce can create incentives for more business opportunity for investment.

The endogenous factors affecting development sector (Figure 3: top right) include: gross domestic product (GDP), political stability, corruption, income and prevailing social condition of a country. One of the responsibilities of government is to ensure stable economy, growth and development. Subsequently, governmental policies and regulations in a country can affect investment, employment and economic growth. Political instability for instance, can contribute to a country’s underdevelopment through adverse effects on worker productivity, income distribution, disruption in healthcare delivery and negatively influences economic performance. Similarly, given the evidence that socioeconomic status highly affects health and health affects income, higher economic development, can translate into higher incomes for workers leading to improvement in the country’s healthcare development and infrastructure, people’s well-being, and the environment.

**Figure 4:**
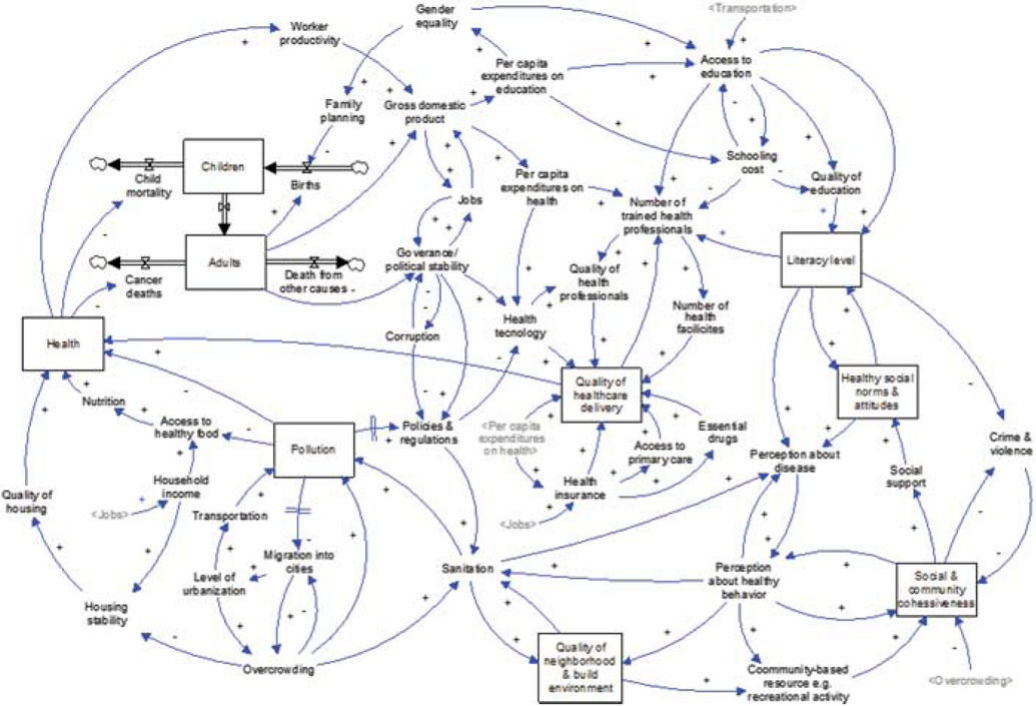
Causal framework of social determinants of health and global cancer disparities

The healthcare system sector (Figure 4: bottom left) captures factors contributing to increased health and life expectancy of the population. A strong healthcare infrastructure is essential to meeting the healthcare needs of the population as well as reducing high costs associated with premature preventable deaths. Lack of adequate healthcare infrastructure such as essential drugs and access to primary care and specialty care are the major barriers to effective care delivery in most developing countries. To effectively meet the growing health needs and reduce health disparities between developed and less developed countries, it is important for governments to invest in modern healthcare technology and implementation of policies that support individual and community health.

Neighborhood and the built environment subsystem (Figure 3: bottom right) shows prevailing factors in the built environment and their subsequent contribution to global health inequalities in healthcare and cancer care. The rising rates in cancer in developing countries have been attributed in part to lifestyles similar to the developed countries [4-6]. The model shows how the built environment can have profound effects on the health of the population. Access to transportation will ease travel time and access to health care. Also, low crime rates for instance will encourage people to lead more active lifestyles. However, lack of community resources, high pollution and crime rates are likely to influence physical inactivity and exacerbates diseases like cancer.

### 3.2 A System Dynamics Framework and Feedback Structure for Global Cancer Disparities

Based on the review, we synthesized the results in the form of a system dynamics causal map, specifically a hybrid diagram that uses both the stock and flow conventions of system dynamics and a causal loop diagram (see Figure 4). Figure 4 has 8 major accumulations or stock variables shown in boxes as: children and adult population, overall population health (health), pollution, quality of healthcare delivery, quality of neighborhood and built environment, social and community cohesiveness, healthy and social norms and attitudes, and literacy level which shows a high level diagram capturing the major feedbacks in the system. The population is represented as a stock and flow structure where the boxes represent the current stocks of the main system and the double lines (“pipes”) with two triangles (“valves”) represent the flows or rates of change to the stocks [11]. For example, the stock of children increases with births and decreases with child mortality and children aging into adulthood. The rest of the diagram (Figure 4) is a causal loop diagram where the other key stocks are also drawn with boxes, but the flows represented the rates of change to the stocks are excluded to improve the readability of the diagram.

The 8 stocks are related through a set of hypothesized causal mechanisms (single lines with arrowheads showing the direction and polarity of the cause-effect relationship) identifying the potential role of multiple interacting feedback loops as explanations for trends in global health cancer disparities. The double lines crossing the causal links represent significant delays between causes and effects. The plus signs mean that increasing the cause variable increases or adds to the effect variable with everything else being held constant. In contrast, minus signs mean that increasing the cause variable decreases or subtracts from the effect variable with everything else held constant [38].

There are two major types feedback loops in a system: reinforcing and balancing loops. Reinforcing feedback loops amplify or accelerate the rate of change. For instance, the larger worker productivity, the greater the growth of GDP, which will lead to more availability of jobs, leading to higher household income for workers. Higher incomes then enable greater access to healthy food which improves nutrition, health, and “feeds back” to increasing worker productivity to form a reinforcing feedback loop (see Figure 4). Reinforcing feedback loops can generate “virtuous cycles” and “vicious cycles”. The feedback loop just described can work in the favorable direction, but the *same* feedback loop or structure can also operate in the unfavorable direction as a “vicious cycle”.

Balancing feedbacks counteract and oppose change. For example, migration into cities increases overcrowding which can contribute to pollution, which eventually slows migration into cities (see Figure 4). This balancing loop counteracts the initial increase of migration.

## 4 Discussion

### 4.1 Children, Adult and Overall Population Health

One of the greatest public health achievements over the last two decades is the decline of childhood mortality in developing countries [10]. These improvements can be attributed to vaccinations against childhood infections, antibiotics against a wide range of bacterial infections, oral rehydration therapy for diarrhea, and in some places, generally improved living conditions [10]. Nonetheless, the growing burden of the cancer epidemic in low-and middle-income countries, due to increases in life expectancy and behavioral life style changes, means more will eventually die from the disease. An estimated 18% of cancer deaths in low- and middle-income countries can be attributed to smoking [12]. Overall, about one-third of cancers in low- and middle-income countries are preventable, considering risk factors such as tobacco use, unhealthy diet, alcohol consumption, sedentary behaviors, pollution and infectious agents [1,12].

The health of a nation’s population is considered the fundamental importance to a country’s well-being and ability to prosper economically. Protecting the health of the population contributes to society by enhancing an individual’s current productivity, as well as that of future generations. If an individual’s health is compromised, there will be serious negative consequences for families, communities and the entire process of economic and social development. Sadly, faced with competing health priorities, most low- and middle-income countries lack the resources to address the challenge of cancer [13]. While more than 80% of the global cancer burden occurs in less to middle-income counties, less than 5% of global health spending is on cancer [13]. This problem is compounded by varying degree of structural disparities inherent in stigmatization, poverty and lack of political will, resulting in lack of access to quality healthcare cancer control program [13,14]. Considering that the vast majority of the population in low-and middle-income countries cannot afford the cost of cancer treatment, a diagnosis of cancer does nothing but contributes to the vicious cycle of poverty.

In the causal map we highlighted the relationship between health and productivity and its effect on poor health, worker’s productivity and earning power, as well as its contribution to a cycle of poverty, health and human capital outcomes across generations (see Figure 4). Lower productivity of workers can lead directly to poverty trap whereby reduced output of mothers and fathers due to poor health also leads to poverty and, subsequently, to a worsening of health outcomes for their children. For example, adverse health events may result in child labor substituting for the work of their parents, potentially lowering children’s educational attainment and their own future productivity. Parental illness or death will not only limit productivity in the labor market, but also impact the ability of parents to care for their children; greatly increasing the risk that adverse health events will have long-lasting consequences [15]. The expectations for a short life span will also reduce savings, and thus investment in physical capital. Secondly, disease and early mortality among the children themselves have adverse intertemporal effects. Illness and malnutrition among children reduce the incentives for parents to invest in their education. This is manifested in both delayed entry into school, as well as early exits. Disease and hunger also diminish cognitive functioning and the ability to learn, thus diminishing the quality of health and literate population (see Figure 4).

### 4.2 Pollution

For many years, air pollution was considered a major problem of environmental health. Several studies have reported an association between atmospheric pollution and diseases such as cardiovascular, respiratory in terms of high mortality and morbidity [16,17,39-47]. A study by Cohen et al. [17] on the global burden of disease due to outdoor air pollution found that outdoor particulate matter (PM) air pollution is estimated to be responsible for about 3% of adult cardiopulmonary disease mortality; about 5% of trachea, bronchus, and lung cancer mortality; and about 1% of mortality in children from acute respiratory infection in urban areas worldwide. This amounts to about 0.80 million (1.2%) premature deaths and 6.4 million (0.5%) lost life years. In the United States studies [18,19] have also reported a link between air pollution cancer risks among urban residents. Similarly, in Europe Barbone et al. [20] also indicated an increased risk for lung cancer among city residents living in the most polluted areas than those living in less polluted neighborhood. In spite of these findings, the debate surrounding the effect of environmental air pollution remained unresolved until recently when air pollution was officially classified as carcinogenic to humans by WHO experts [2]. The International Agency for Research on Cancer (IARC) concluded that there is sufficient evidence that exposure to outdoor air pollution causes lung cancer, and an increased risk of bladder cancer. “The air we breathe has become polluted with a mixture of cancer-causing substances,” says Dr. Kurt Straif, Head of the IARC Monographs Section [2]. While the levels of pollution vary between/within countries as well as urban and rural areas, its effect is expected to be greater in less developed countries especially Africa and Asia due to massive undergoing economic development resulting in rapid levels of urbanization air pollution [48, 49].

Even though increased economic growth and development is associated with increased urbanization, the majority of urban population growth in less developed countries occurs among people living in poverty and results in growth of slums, overcrowding and unsanitary conditions. In the model, we argued that pollution negatively affects the overall health of pollution that will result in reduced worker productivity, gross domestic product (GDP), per capita expenditure on health, access to education, quality education and general literacy level (see Figure 4). Available evidence suggests that economically deprived communities have higher risk for related air pollution morbidity and mortality due lack of access to healthcare services, poorer nutrition and other factors [48,49]. Air pollution could therefore exacerbate the deplorable health conditions of in poor regions of the world. In addition, Gouveia et al. [21] and Jerrett et al. [22] have all argued that low level of education and income are associated with increased related air pollution health effects. These findings have therefore underscored the importance of social determinants to global cancer disparities.

According to the United Nations Population Fund (UNFPA), about half of the world’s population currently lives in towns and cities. However, by 2030 this number is expected to increase to 5 billion people with Africa and Asia experiencing most of these transformations [51]. While urbanization has the potential to lead to economic development, it can also lead to rise in slums, poverty, disparities and unhealthy life styles. In the model we argued that migration to the cities will increase the level urbanization which in turns will lead to overcrowding and its health hazards (see Figure 4).

### 4.3 Quality of Healthcare Delivery

While global concern about health inequities is growing, very little attention has been focused on the rapidly increasing toll of cancer in developing countries. As noted by the International Union against Cancer 2010 (UICC), the odds of surviving cancer based on the type of treatment one receives, including basic palliative care are strongly correlated with place of residence [23]. Whereas in the United States the five-year survival rate for patients with breast cancer is 84%, in the Gambia, it is only 12% [24].

Further, advances in biomedical technology, resulting in new improved cancer management have contributed to a considerable decrease in cancer mortality rates in most developed countries [25]. Cancer, once considered a disease of affluence, has become a death sentence in the developing world due to the absence of healthcare services and cancer drugs. According to the Institute of Medicine, cancers in low-middle income countries are diagnosed much later. It is estimated that up to 80% of cancers are detected at late stage hence, incurable by the time they are discovered [10]. Limited access to health services, poverty, lack of insurance, primary care, unhygienic practices are major factors contributing to the widen disparity gap between advanced and less developed nations.

It is a fact that every country has its own specific cancer burden features, risk factors, culture, health system, and available financial and human resources. Consequently, the level and degree of disparity also differ within and between countries. In the developed Organization for Economic Cooperation for Development (OECD) countries access to healthcare services is universal, but inequalities in health status have been shown to be related to income and other socio-economic factors [26,27]. In the model, we indicated that social determinants of health delivery is influenced by the effects of other social factors like per capita expenditures on health, jobs opportunity, health insurance, access to primary number of trained health professionals, number of health facilities, quality of health professions, health technology among others (see Figure 4).

The model on Figure 4 further highlights some of the major feedback structure that show the relationship between quality of healthcare delivery and general health and well-being as well as the ultimate impact on global cancer disparities.

### 4.4 Quality of Neighborhood and Built Environment

The neighborhood where we live and its environs can influence our health, depending on the factors such as the community design, recreational activity, quality of housing, schools, access to medical care and food, transportation, and air and water pollution [28,53]. Owen, Obregon and Jacobsen [29], analyzed the impact of geographic access to health services in rural Guatemala and indicated that the poorest communities in Alta Verapaz have the least geographic access to health center. Another study conducted by Campbell and colleagues [30] in Scotland on rural factors and cancer survival revealed that increasing distance from a cancer center was associated with greater chance of the patient being recorded as a death certificate only (*DCO – patients for whom only the death certificate provides notification to the cancer registry*) case for stomach, breast and colorectal cancers. In Taiwan, Chang et al. [31] suggested that the existence of inequality in healthcare resources like available diagnostic tools and treatment technologies in rural areas are contributing to higher risk of nasopharyngeal cancer in rural areas. While regional and district hospitals have CT scans to help staging rural hospitals do not have these services and where they are available, they may be too expensive for patients to afford it. Health is also shaped by social relationships. For instance neighborhood where residents express mutual trust have been reported to have a lower crime and homicides rates [32].

According to Maller et al. [33] health cannot be separated from other social determinants. The interplay between people and their environment constitutes the basis for a socio-ecological approach to health and well-being [33]. Using the casual mechanism the model explained how neighborhood and built environment contribute to cancer disparities. For instance, friendly and safe environment would encourage residents to engage in physical activity. Through regular physical activities, neighbors become more engaged leading to more social and community cohesiveness and support. Interaction with family, friends, neighbors and co-workers would increase a sense of identity and eliminating stigma and unhealthy norms and attitudes about cancer and finally improved understanding about healthy life styles (see Figure 4).

### 4.5 Social Norms and Community Cohesiveness

Social norms are sets of rules that define appropriate and inappropriate values, behaviors, beliefs and attitudes within a group. Norms are created for several reasons: (1) to maintain cohesive order in a group or society as a whole, (2) to define boundaries of appropriateness and (3) to create a collective sense of community [34]. Social norms and cohesiveness also provide a model for understanding human behavior that has important implications for health and well-being.

To eliminate health disparities, it is important to understand the influence of culture on each society’s beliefs, attitudes and public health practices [35]. For example, gender and cultural norms and values, in some society may give rise to gender inequalities. In some cultures a woman cannot receive needed healthcare from a male because norms in her community may perceive that as a taboo. Lack of cohesion is also associated with higher levels of crime, fear of crime and antisocial behavior. In our model framework, we indicated that a higher level of social cohesion may also provide more social support and mutual respect, and influence beliefs, practices and perceptions about cancer and other related health (see Figure 5).

Similarly, healthy social norms and community cohesiveness can facilitate support to promote higher literacy level in the community, which can also enhanced people’s perception about cancer and other diseases, as well as their perception to engage in healthy behavioral life styles changes like participating in recreational activities leading to more healthy norms in the community (see Figure 5).

### 4.6 Literacy Level

Formal education is not an end to health literacy, but an important element for economic development. However, the fact remains that illiteracy is still high and costing the world lots of money [36,37]. According to the World Literacy Foundation, illiteracy costs the global economy almost USD $1.5 trillion dollars each year [37]. Although the severity of illiteracy varies between developed and less developed countries, its effects are similar, including inability to have employment and low income earning jobs and potentially remaining in poverty. Education can leads to improved health outcomes, in that more educated individuals make better informed health decisions [32,36]. For example, a low literate society is characterized by high level of crime and violence, low social and community cohesiveness which will intend leads to low social support and unhealthy social norms and attitudes. The causal map framework in figure highlights the importance of literacy, healthy social norms and social norms and community cohesiveness and their contribution to global cancer disparities. For instance, higher level of literacy will lead to improved personal hygiene practices and healthy life styles and perceptions about diseases and removal of stigma on those who suffered from it (see Figure 4).

## 5 Conclusions

Addressing these determinants of health requires effective dynamic approaches to improving global cancer health. Using causal loop diagraming, we provided a synthesis of the research from a system dynamics perspective by identifying underlying factors of global cancer disparities, how they are interrelated in the system and the consequences on health and well-being. Despite the challenges of eliminating health disparities, we believe the application of a systems thinking methodological approach is necessary to provide new understanding on how global development trends combined with global health efforts to improve population health could shift cancer disparities and burden associated with the disease. This is necessary because public health challenges are particularly complex, because they are often intertwined with much larger organizational, social, environmental, and cultural problems. Understanding these issues is essential to enhance the design and implementation of programs and policies to meet the needs of each specific environment. Subsequently, it is essential to approach global health issues from a broad systems perspective in order to have a comprehensive understanding of how factors are interconnected and interacting within the whole system. For future work we propose focusing on developing a quantified computer simulation model to identify and assess potential leverage points for intervention.
